# Immune checkpoint blockade in HIV

**DOI:** 10.1016/j.ebiom.2022.103840

**Published:** 2022-02-02

**Authors:** Celine Gubser, Chris Chiu, Sharon R. Lewin, Thomas A. Rasmussen

**Affiliations:** a Department of Infectious Diseases, The University of Melbourne at the Peter Doherty Institute for Infection and Immunity, 792 Elizabeth Street, Melbourne, Victoria 3000, Australia; b Victorian Infectious Diseases Service, Royal Melbourne Hospital at the Peter Doherty Institute for Infection and Immunity, Melbourne, Australia; c Department of Infectious Diseases, Alfred Hospital and Monash University, Melbourne, Australia; d Department of Infectious Diseases, Aarhus University Hospital, Aarhus, Denmark

**Keywords:** HIV, Immunotherapy

## Abstract

Antiretroviral therapy (ART) has dramatically improved life expectancy for people with HIV (PWH) and helps to restore immune function but is not curative and must be taken lifelong. Achieving long term control of HIV in the absence of ART will likely require potent T cell function, but chronic HIV infection is associated with immune exhaustion that persists even on ART. This is driven by elevated expression of immune checkpoints that provide negative signalling to T cells. In individuals with cancer, immune checkpoint blockade augments tumour-directed T-cell responses resulting in significant clinical cures. There is therefore high interest if ICB can contribute to HIV cure or remission by reversing HIV-latency and/or drive recovery of HIV-specific T-cells. We here review recent evidence on the role of immune checkpoints in persistent HIV infection and discuss the potential for employing immune checkpoint blockade as a therapeutic approach to target HIV persistence on ART.

## Introduction/Aims of immune checkpoint blockade in HIV

Antiretroviral therapy (ART) potently suppresses HIV replication and has greatly improved prognosis for people with HIV (PWH) but it is no cure. The main reason ART is unable to cure HIV is the persistence of HIV in a latent form in long-lived and proliferating CD4+ T-cells from which virus rebounds if ART is stopped.^1^ Achieving durable control of HIV in the absence of ART is likely to be mediated by potent CD8+ T-cell effector function, similar to what is observed in HIV elite controllers.^2^ However, HIV infection is characterised by immune exhaustion, driven by increased expression of immune checkpoints,^3^, ^4^, ^5^, ^6^ which persists even on ART^7^ leading to a state of immune dysfunction with impaired cytolytic activity.^8^

Immune checkpoints make up a network of receptors involved in maintaining a balance between T-cell activation and autoimmunity by providing co-inhibitory or co-stimulatory signalling that modify the quality and duration of the T-cell effector response.^9^ Programmed cell death 1 (PD-1) and cytotoxic T-lymphocyte associated protein 4 (CTLA-4) are the best studied pathways, but other receptors of significant interest in both infectious disease and cancer include lymphocyte activation gene 3 (LAG-3), T-cell immunoglobulin and ITIM domain (TIGIT) and T-cell immunoglobulin and mucin-domain containing-3 (TIM-3). Therapeutic blockade of PD-1, PD-ligand1 (PD-L1) and CTLA-4 by monoclonal antibodies has demonstrated clinical efficacy in cancer by enhancing tumour-specific T-cell function,^10^^,^^11^ but monoclonal antibodies to multiple other immune checkpoints including LAG-3, TIM-3 and TIGIT are currently under active clinical development.^12^

**Figure 1:**
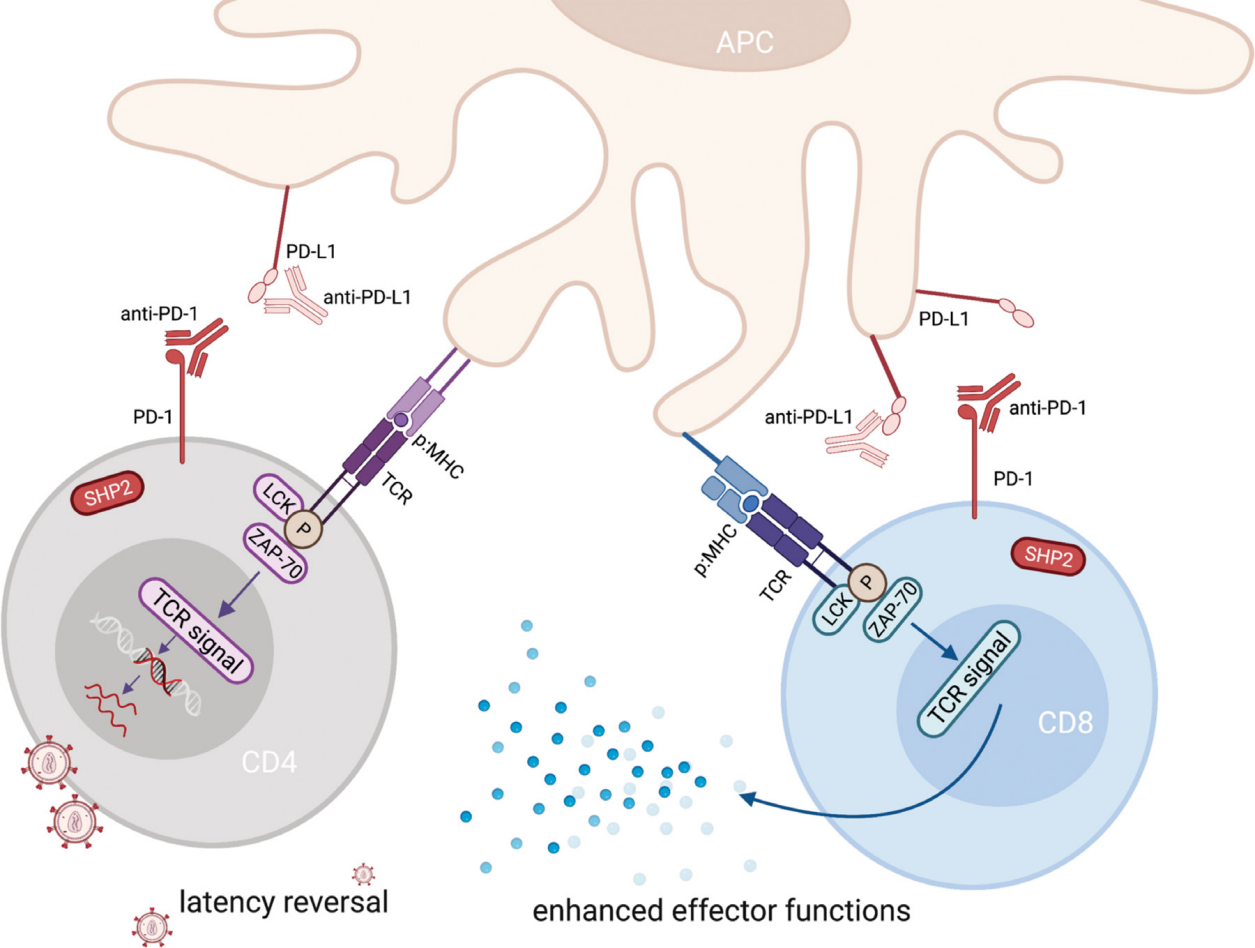
*Dual Role of ICB in HIV*. Immune checkpoint antibodies have two distinct effects in the setting of HIV on ART. On the one hand they activate HIV expression in latently infected CD4+ T-cells and on the other hand they enhance HIV-specific CD8+ T-cell function. PD-1: Programmed death-1; PD-L1: Programmed death ligand-1; SHP2: Src homology 2 domain-containing tyrosine phosphatase 2; TCR: T-cell receptor; LCK: lymphocyte-specific protein tyrosine kinase; ZAP-70: Zeta-chain-associated protein kinase-70; MHC: major histocompatibility complex.

In addition to their role in HIV-associated immune exhaustion, immune checkpoints also identify cells that are enriched for HIV. CD4+ T-cells expressing PD-1, LAG-3 and/or TIGIT have been shown to contain HIV DNA at a higher frequency,^13^, ^14^, ^15^ in particular in cells co-expressing multiple immune checkpoints.^6^^,^^13^ This might be explained by inhibitory signalling during T-cell infection, limiting T-cell activation and favouring transition to latent infection, as was demonstrated in an *in vitro* model.^16^ Immune checkpoint proteins thus contribute to HIV persistence through two major pathways – effects on the virus itself by promoting latent infection and by impairing cytotoxic function of HIV-specific T-cells. Given this dual role, immune checkpoint inhibitors may similarly have two distinct effects when given to PWH on ART: (1) they may activate HIV expression in latently infected CD4+ T-cells thereby exposing these to immune recognition and/or viral-induced apoptosis; and (2) they may reinvigorate exhausted T-cells, thereby enhancing HIV-specific T-cell function (Figure 1).

The potential role of immune checkpoint antibodies in targeting HIV persistence on ART depends on their capacity to achieve these effects as well as their safety profile in PWH on ART without cancer. Both outcomes may be optimised by alternative dosing strategies, by combination approaches with other immunomodulators, latency-reversing agents or vaccines, by identifying pathways associated with a favourable virological response, and/or by delivering immune checkpoint blockade in the context of antigen co-stimulation. A detailed understanding of the underlying biology of immune checkpoints is fundamentally important to assessing how interrupting these pathways might be relevant for curative strategies in HIV and potentially other chronic viral infections. This is driven forward by active research across *in vitro, ex vivo* and *in vivo* models in HIV as well as emerging data from clinical trials in PWH on ART. We here review data regarding the role of immune checkpoints in persistent HIV infection with a particular focus on recent and emerging findings from clinical trials in PWH and discuss the potential for employing immune checkpoint blockade, including combined blockade of multiple checkpoints, as a therapeutic approach to achieve durable control of HIV in the absence of ART.

## T-cell exhaustion in cancer and chronic infection

Common features of cancer and chronic infections are persistent exposure to antigen and the development of dysfunctional or exhausted effector T-cells.^17^ While multiple studies have shown tremendous success in treating cancers with antibodies that block inhibitory receptors on T-cells (immune checkpoint blockade, ICB) it is unclear why some individuals respond and others do not. The anti-tumour clinical response to PD-1 blockade in patients with malignancies^18^^,^^19^ was recently shown to rely on precursor exhausted T-cells (Tpex), a subpopulation of CD8+ T-cells that exhibit features of exhaustion, such as PD-1 expression, but also display memory T-cell characteristics,^20^, ^21^, ^22^, ^23^ including expression of the transcription factor T-cell factor 1 (TCF-1). TCF-1 is crucial for the development of memory CD8+ T-cells.^24^ Tpex were found to be responsible for the proliferative burst and increased CD8+ T*-*cell effector functions after blocking PD-1^25^, ^26^, ^27^ whereby they replenish the population of terminally differentiated exhausted T-cells (Tex), which are essential for ongoing immune control. A higher frequency of Tpex cells was associated with better anti-tumour responses and improved patient survival,^18^^,^^19^ and a higher ratio of Tex cells to tumour burden predicted an enhanced clinical response.^11^

In the setting of HIV, CD8+ T-cells from elite controllers, individuals who can control HIV replication to undetectable levels in the absence of ART, were shown to express higher levels of TCF-1 compared to non-controllers,^28^ suggesting that TCF-1 may have a direct role in regulating the expansion capacity of HIV-specific CD8+T-cells. However, it remains to be proven if HIV-specific Tpex can be targeted with ICB in PWH on ART leading to recovery of polyfunctional HIV-specific T-cells.

Another mechanism by which HIV-specific CD8+ T-cells from elite controllers have been reported to evade exhaustion was linked to HLA- B*27 and HLA-B*57 alleles. Upon antigen recognition HLA-B*27– or HLA-B*57–restricted HIV-specific CD8+ T-cells showed a deficiency in TIM-3 upregulation, allowing these cells to evade TIM-3:Gal-9 mediated regulatory T-cell (Treg) suppression.^29^

## Role of immune checkpoints in HIV and pre-clinical data on blocking these pathways

In cancer and chronic infection, the understanding of immune checkpoints and their targeting is a significant milestone toward reversing T cell exhaustion. In this review we have put an emphasis on immune checkpoints assessed in preclinical studies and recent findings from clinical trials in HIV. While other immune checkpoints like CD39, V-domain Ig suppressor of T cell activation (VISTA), CD244 (2B4) and CD160 have all been associated with T-cell exhaustion in HIV infection, there is little preclinical and no clinical data on targeting these receptors in the context of HIV infection.

## PD-1

**Figure 2:**
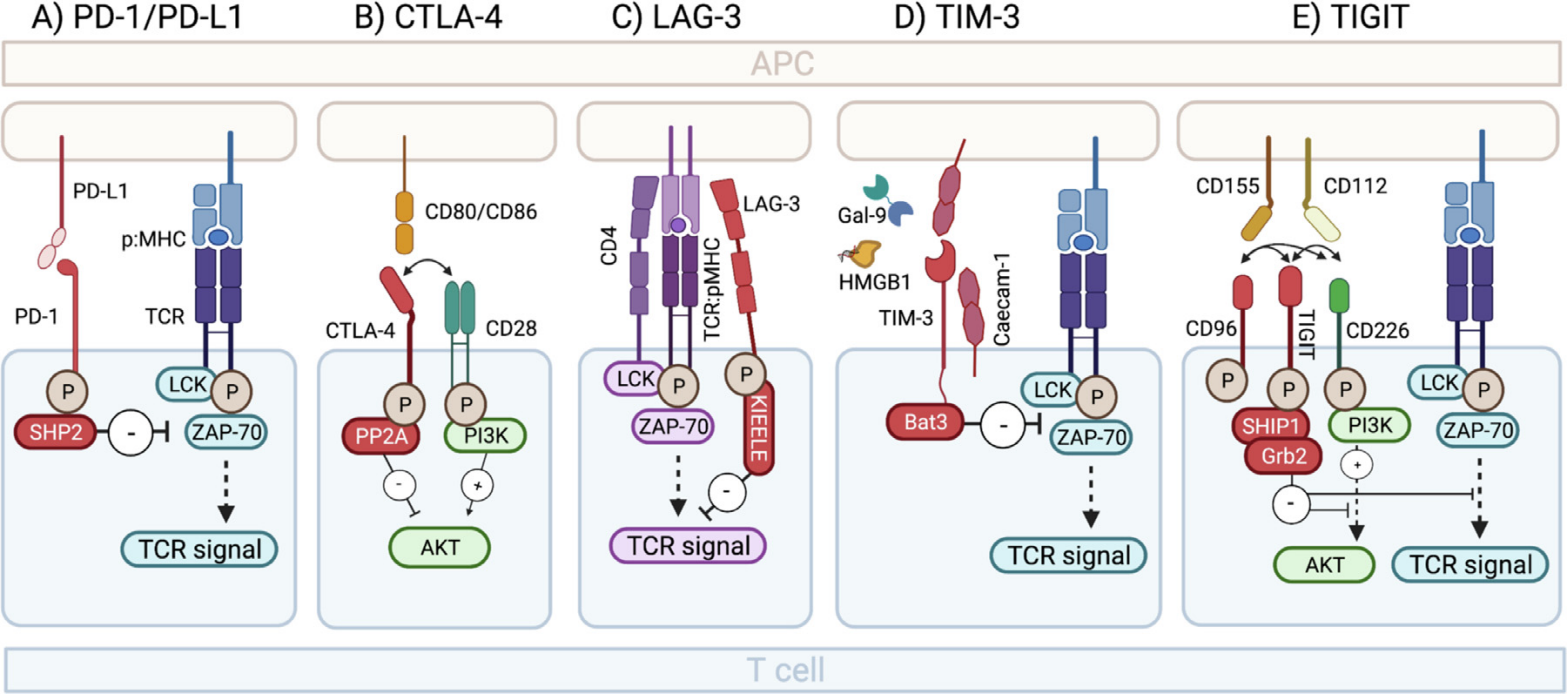
*Immune checkpoint inhibition: Receptor/ligand interaction and signaling***. (A)** PD-1-PD-L1/PD-L2 interaction signals through the protein tyrosine phosphatase SHP-2 (Src homology 2 domain-containing tyrosine phosphatase 2), which dephosphorylates kinases and blocks proximal TCR signal transduction. (**B)** Upon binding CD80/CD86 the cytoplasmatic tail of CTLA-4 transduces a signal through the protein phosphatase 2A (PPA2) to inhibit phosphorylation of Akt and thereby interfering with IL-2 production, cell cycle progression and proliferation. (**C)** LAG-3 is believed to signal through its unique KIEELE motive to transduce antiproliferative signals from the TCR. However, the intracellular proteins that bind the KIEELE motif and the signalling pathways further downstream are still not known. (**D)** Upon Caecam-1 and Gal-9/TIM-3 triggering, Bat3 gets released form the cytoplasmatic tail of TIM-3 and allows binding of SH2 domain containing Src kinases like LCK and ZAP-70 which subsequently block TCR signalling. (**E)** Upon ligand interaction, TIGIT becomes phosphorylated and recruitment of SHIP1 (SH2 domain containing inositol-5-phosphatase) and Grb2 (growth factor receptor bound protein 2) lead to blocking of PI3K (phosphatase 3-kinase) and MAPK (mitogen-activated protein kinase) pathways resulting in reduced T-cell activation, proliferation, and effector functions. PD-1: Programmed death-1; PD-L1: Programmed death ligand-1; CTLA-4: Cytotoxic T-lymphocyte associated protein 4 (CTLA-4); Lymphocyte activation gene 3; TIGIT: T-cell immunoglobulin and ITIM domain; TIM-3: T-cell immunoglobulin and mucin-domain containing-3 (TIM-3); TCR: T-cell receptor; LCK: lymphocyte-specific protein tyrosine kinase; ZAP-70: Zeta-chain-associated protein kinase-70; MHC: major histocompatibility complex.

PD-1 is a co-inhibitory receptor expressed on the surface of activated T-cells and binds to PD-L1 and PD-L2, which are expressed on the surface of antigen presenting cells (APCs) (see Figure 2 and Text box 1).

**Text box 1:** Additional information on IC receptors/ligand interactions.

**PD-1** PD-1 (also known as CD279) binds to PD-L1 (also known as CD274) and PD-L2 (also known as CD273) which are expressed on dendritic cells, monocytes, B cells and macrophages. Targeting the PD-1 receptor or the PD-L1/PD-L2 ligand with monoclonal antibodies blocks the inhibitory receptor-ligand interaction and takes the “breaks” off the activating TCR signals (Figure 2.) **CTLA-4** CTLA-4 (also known as CD152) is a homologue of the co-stimulatory molecule CD28 and like CD28 binds to CD80 (also known as B7–1) and CD86 (also known as B7–2) expressed on APCs, but with higher affinity and therefore outcompetes the co-stimulatory signal (Figure 2). **LAG-3** LAG-3 (also known as CD223) is a CD4 homolog that binds to the major histocompatibility complex (MHC) class II with superior affinity. LAG-3 is upregulated on activated T-cells, Tregs and a subset of NK cells.^45^ Stimulating LAG-3 deficient T-cells *in vitro* and *in vivo* resulted in their uncontrolled expansion^89^^,^^90^ consistent with LAG-3 being a negative regulator of T-cell expansion (Figure 2). However, the effect on CD8 and NK cells is likely indirect as neither of these express CD4 or bind MHC-II. This could be through Treg mediated suppression as Tregs constitutively express high levels of LAG-3^91^ and were shown to inhibit DC maturation,^92^ thereby reducing immune activating stimuli and suppressing T-cell activation. **TIM-3** TIM-3 (also known as Hepatitis A virus cellular receptor 2, HAVCR2) has multiple ligands. Apart form Gal-9 it interacts with the high mobility group protein B 1 (HMGB1)^93^ that binds DNA released form dying cells and facilitates delivery to Toll-like receptors (TLRs). TIM-3 can block this process by binding to HMGB1 and thereby suppress innate immune activation. Caecam-1 was recently identified as a crucial component in the co-inhibitory function of TIM-3.^94^ Caecam-1 is expressed on the surface of exhausted T-cells together with TIM-3 and while in *cis* it acts as a stabilizer for the TIM-3 glycoprotein, the *trans* interaction drives inhibitory Tim-3 function. It is uncertain, however, if binding TIM-3 by Caecam-1, Gal-9 or both together differentially impact TIM-3 function (Figure 2). **TIGIT** TIGIT is a receptor of the Ig superfamily and binds to the ligands CD155 (also known as poliovirus receptor (PVR) or Necl-5) and CD112 (also known as PVRL2, nectin-2) expressed on APCs, T-cells and tumour cells. The co-stimulatory molecule CD226 (also known as DNAM-1) and co-inhibitory molecule CD96 (also known as Tactile) compete for the CD155 and C112 ligands. Similar to the CTLA-4-CD80/86-CD28 setup, TIGIT binds its ligands with superior affinity therefore favouring effector cell inhibition over stimulation.^95^ CD226 is important for effective tumour-specific CD8+ T-cell responses and it was recently shown that T-cells expressing high CD226 expression maintain effector functions in response to TCR triggering despite co-expression of negative receptors, like PD-1.^96^

In HIV infection, PD-1 overexpression on both CD4+ and CD8+ T-cells was shown to correlate with increased disease progression and higher HIV viral load.^4^^,^^30^ Apart from persistent T-cell receptor (TCR) stimulation, *in vitro* studies also indicate that the negative regulatory factor (Nef) can induce expression of PD-1 in HIV-1-infected cells by activating the p38 pathway.^31^

It was shown more than 15 years ago that during chronic HIV infection, increased expression of PD-1 on HIV-specific CD8+ T cells is associated with reduced effector functions and that blocking PD-1 could enhance HIV-specific CD8+ T-cell survival, proliferation, and reinvigorate effector functions upon T cell receptor (TCR) stimulation with cognate antigen.^5^ In a study where simian immunodeficiency virus (SIV)-infected rhesus macaques were administered anti-PD-1 ten days prior to ART initiation and again at 26–30 weeks following ART, the authors found faster viral suppression in plasma, enhanced CD8+ T-cell function and improved CD4+ T-cell reconstitution in the gut leading to a significant delay in viral rebound and a reduction of the viral setpoint following ART interruption.^32^

Consistent with the enrichment of HIV in PD-1+CD4+ T-cells,^13^, ^14^, ^15^ it was also shown that blocking PD-1 *in vitro* or *ex vivo* can induce or enhance activation of latent HIV.^16^^,^^33^^,^^34^ However, in a recent study in ART-treated SIV-infected macaques, PD-1 blockade, alone or in combination with a TLR7 agonist, showed no impact on viral rebound kinetics following ART interruption and no impact on the frequency of latently infected cells.^35^

Interestingly, PD-1 blockade during SIV-vaccination substantially improved protection against SIV infection of macaques and protective SIV-specific T-cell responses were sustained for more than 42 weeks after the first immunisation.^36^ Collectively, these studies indicate an immune-enhancing effect of PD-1 blockade in the setting of HIV infection.

## CTLA-4

CTLA-4 is a potent negative regulator of immune responses and is expressed on both activated T-cells and regulatory T-cells (Tregs).^37^ It binds to CD80 and CD86 expressed on APCs (see Figure 2 and Text box 1).

During untreated HIV infection, CTLA-4 expression is upregulated on HIV-specific CD4+ T-cells with only modest and slow reduction in expression levels upon starting ART.^3^ A study in SIV-infected macaques on ART identified a potential viral reservoir in lymph node CTLA-4+PD-1- memory CD4 T-cells as these cells contained high levels of SIV DNA as well as replication-competent and infectious virus.^38^ Additional data imply that viral factors influence CTLA-4 expression. *In vitro* studies showed that Nef-mediated downregulation of CTLA-4 in HIV-infected CD4+ T-cells resulted in enhanced interleukin (IL)−2 production and viral replication upon TCR triggering.^39^ This led to the hypothesis that infection of CD4+CTLA-4 + T-cells elicited Nef-mediated concomitant downregulation of CTLA-4, which is believed to produce optimal conditions for viral replication, thereby promoting productive infection and HIV persistence.^39^

Kaufmann et al. showed that increased levels of CTLA-4 expression on HIV-specific CD4+ T-cells are associated with disease progression and a failure to produce IL-2, and that *in vitro* blockade of CTLA-4 could rescue HIV specific CD4+ T-cell function.^3^ In HIV disease progressors it was shown that CTLA-4 upregulation on HLA-B35px and HLA-B53 restricted HIV specific CD8+ T-cells and the expression of CTLA-4 correlated with low proliferative capacity, poor expression of cytotoxic molecules and decreased cytokine production of HIV-specific CD8+ T-cells.^40^ A study in SIV-infected rhesus macaques treated with ART similarly showed that CTLA-4 blockade was associated with an increase in SIV-specific CD4+ and CD8+ T-cell effector function and a decrease in viral RNA levels in lymph nodes, thus indicating a role for anti-CTLA-4 in augmenting anti-SIV immunity.^41^ In contrast, another study using the same virus (SIV_mac251_) in rhesus macaques found that CTLA-4 blockade during primary infection increased both T-cell activation and viral replication, and that viral suppression following ART initiation was blunted in monkeys receiving anti-CTLA-4.^42^ More recently, Harper et al. investigated anti-PD-1 and anti-CTLA-4, alone or in combination, in SIV infected macaques on long-term ART and showed that although dual blockade was more effective in expanding effector memory T-cells and induced latency reversal at higher levels compared to anti-PD-1 alone, none of the tested interventions enhanced SIV-specific CD8+ T-cell function or virological control following ART discontinuation.^43^

Finally, a murine study indicated a potential adjuvant role of anti-CTLA-4 as CTLA-4 blockade during HIV immunization in mice led to increased CD4+ T-cell activation, expansion of HIV specific follicular helper T-cell (Tfh) cells, altered HIV specific B-cell responses and significantly increased anti-HIV antibodies with higher avidity and antibody-dependent-cellular cytotoxicity (ADCC) capabilities.^44^

## LAG-3

LAG-3 binds to the major histocompatibility complex (MHC) class II and is upregulated on activated T-cells, Tregs and a subset of NK cells^45^ (see Figure 2 and Text box 1).

There is still limited data on LAG-3 and LAG-3 blockade in HIV although one study showed enrichment of HIV in LAG-3+CD4+ T-cells.^13^ Also, HIV infection increased LAG-3 expression in both peripheral blood and lymph node CD4+ and CD8+ T-cells, correlated with HIV plasma viral load and disease progression,^46^^,^^47^ whereas *ex vivo* blockade augmented HIV-specific CD4+ and CD8+ T-cell responses.^46^

## TIM-3

TIM-3 is expressed on T-cells and innate immune cells like DCs, NK cells and monocytes and binds to galectin-9 (Gal-9) which triggers inhibitory signaling and can induce cell death of TIM-3 expressing T-cells^48^ (see Figure 2 and Text box 1, 2).

**Text box 2:** Additional IC ligands and their role in HIV.

**Gal-9** is a member of the galectin family of animal lectins and binds to the TIM-3 receptor. It has a wide range in biological properties and its role in the context of HIV is complex. Gal-9 is upregulated on NK cells in PWH^97^ and on CD4+ and CD8+ T-cells with impaired T-cell effector function.^98^ Gal-9 was also shown to be rapidly released during acute HIV infection with high levels remaining in the circulation even upon viral control with a positive correlation between plasma Gal-9 levels and HIV viral load.^99^ Furthermore a recent report demonstrated the ability of Gal-9 to reactivate latent HIV-1 in a jurkat T-cell line.^52^ This finding is consistent with a recent study showing Gal-9 shedding by neutrophils can activate T cells via binding to CD44 which leads to T cell activation in PWH.^100^ **CD155** is a ligand of the TIM-3 receptor and was shown to be upregulated on CD4+ Tfh cells that reside in the lymph node, a major site of HIV persistence.^53^^,^^56^^,^^101^ It was suggested that HIV can directly upregulate CD155 cells through a Vpr-dependent mechanism,^102^ but newer studies challenged these observations.^103^

In PWH the frequency of TIM-3+ CD8+ and TIM-3+ CD4+ T-cells were both positively correlated with HIV viral load and inversely with absolute CD4 T-cell counts.^49^ Recent *in vitro* studies suggested that Nef-mediated TIM-3 upregulation may in fact activate infected cells,^50^ which is somewhat counterintuitive but highlights the complex biology that exhaustion markers are also markers of T-cell activation. In contrast, the Vpu protein expressed late in the viral replication cycle was shown to downregulate TIM-3, possibly to facilitate viral release.^51^

In HIV progressors, TIM-3 upregulation in HIV-specific CD8+ T-cells was associated with reduced effector functions. In some individuals, this could be reduced upon ART and blocking TIM-3 interaction resulted in reinvigoration and restoration of CD8 effector functions *ex vivo*^49^ implying that neutralizing TIM-3 may have a role in approaches to eliminate the HIV reservoir.^52^

## TIGIT

TIGIT is a co-inhibitory receptor that is specifically expressed on activated T-cells, memory T-cells, Tregs, NK cells and Tfh cells^53^ and has multiple complex ligand interactions that result in reduced T-cell activation, proliferation and effector functions^54^^,^^55^ (see Figure 2 and Text box 1, 2).

TIGIT was shown to be upregulated on CD8 T-cells during HIV infection despite early initiation of ART, and almost all HIV specific CD8+ T-cells from PWH express TIGIT.^6^^,^^56^ Increased TIGIT expression on CD4+ T-cells correlated with the frequency of HIV DNA, and TIGIT co-expression with either PD-1 or LAG-3 marked cells with substantially higher levels of HIV DNA.^13^

A recent study also showed decreased expression of interferon-γ (IFN-γ), tumour necrosis factor-α (TNF-α) and CD107 expression in TIGIT+ NK cells compared to TIGIT− NK cells from PWH.^57^ Nevertheless, introduction of monoclonal antibodies that inhibit the higher affinity TIGIT/CD155 co-inhibitory pathway in favour of the lower affinity CD226/CD155 co-stimulatory pathway remains a rational strategy to re-invigorate HIV-associated T-cell exhaustion to target the HIV reservoir.

## Clinical data of immune checkpoint blockers in PWH

Due to the frequent exclusion of PWH in clinical trials of immune checkpoint antibodies for cancer, there is still limited clinical data on their use in this population, although recent studies including a systematic review reported safety data and anti-tumour response rates comparable to that seen in people without HIV.^58^, ^59^, ^60^ Data from clinical studies regarding the effect on HIV-specific T-cell function, reversal of HIV latency, and the latent HIV reservoir are even more sparse and mostly limited to small studies or case reports of PWH receiving ICB for cancer. These studies have so far mostly focused on the capacity of ICB to activate HIV from latency.

In a person with HIV on ART receiving anti-CTLA-4 followed by anti-PD-1 for melanoma, we previously observed a marked increase in cell-associated and plasma HIV RNA, indicative of latency reversal *in vivo*.^16^^,^^61^ Other case reports of PWH on ART receiving anti-PD-1 or anti-CTLA-4 for cancer have described transient increases in either cell-associated or plasma HIV RNA with or without a decrease in the frequency of latently infected CD4+ T-cells,^62^^,^^63^ whereas others have not seen this effect.^64^^,^^65^ In a small case-series of three PWH on ART receiving anti-PD-L1 for Merkel cell carcinoma or combined anti-PD-1/anti-CTLA-4 for melanoma, we recently reported that ICB led to substantial increases in cell-associated HIV RNA of up to 16-fold relative to pre-treatment levels.^66^ In one individual receiving combined blockade of PD-1 and CTLA-4, there was also a dramatic increase in the frequency of HIV-specific CD8+ T-cells producing IFN-γ, TNF-α, and CD107a expression in response to *gag* stimulation, thus showing the potential to enhance HIV-specific T-cell responses but also indicating that such a favourable response may only occur in a subset of treated individuals.^66^

In a larger prospective study of PWH on ART assigned to anti-PD-1, alone or in combination with anti-CTLA-4, it was found that anti-PD-1 alone did not reverse HIV latency. However, in seven individuals receiving anti-PD-1 in combination with anti-CTLA-4, there was a modest but significant increase in cell-associated HIV RNA as compared to baseline, thus suggesting an enhanced effect on reversing HIV latency with combination ICB.^67^ Only two individuals had large volume blood samples to quantify functional virus, but in both participants there was a substantial decrease in replication-competent HIV, in contrast to those receiving anti-PD-1 alone.^67^ An important limitation of this study is the absence of data on the effect on HIV-specific T cell function during blockade of PD-1 and CTLA-4. These analyses are part of ongoing work.

There are only three published studies of immune checkpoint blockade in PWH on ART without malignancy and two of those were terminated prematurely. One was a dose-escalation study of a monoclonal antibody to PD-L1 (BMS-936,559). Data from the first dose-cohort showed an increase in HIV-specific CD8+ T-cell responses in two of six individuals treated with a single infusion of low-dose (0.3 mg/kg) anti-PD-L1 but no effect on plasma or cell-associated HIV.^68^ The study did not progress to the higher dose-cohorts because of retinal toxicity in a concurrent monkey study and, additionally, one individual in the human trial developed hypophysitis 36 weeks after single low-dose anti-PD-L1(68). More recently, a dose-escalation study of the anti-PD-1 antibody, cemiplimab, in PWH on ART without cancer, was stopped prematurely as possible immune-related adverse events (irAE) occurred in two of four cemiplimab recipients.^69^ One participant developed thyroiditis assessed as probably related to cemiplimab while another had asymptomatic grade 3 hepatitis possibly related to cemiplimab. Both cases fully resolved without therapeutic intervention. An ongoing study is investigating ascending doses of the anti-PD-1 antibody, budigalimab, in PWH in the context of ART interruption (NCT04223804). In another study, ascending dose of the anti-CTLA-4 antibody, ipilimumab, was given to viremic PWH on or off ART to test whether ipilimumab-enhanced immunity might improve virological control.^70^ Assessment of HIV-specific immunity could not be performed due to poor cell viability, but most participants displayed an increase in plasma HIV RNA after ipilimumab dosing, which likely reflected activation of HIV during CTLA-4 blockade.^70^

## Combined blockade of multiple immune checkpoints: is more better?

Superior therapeutic efficacy with combination immune checkpoint blockade was demonstrated in clinical trials of anti-PD-1 and anti-CTLA-4 for melanoma including improved long-term survival but at a cost of higher rates of immune-related toxicities.^71^^,^^72^ Multiple other combinations of immune checkpoint antibodies are now under active clinical investigation in oncology clinical trials with most combinations including an antibody to PD-1 or PD-L1.^12^

PD-1 and CTLA-4 signalling attenuate T-cell activity through separate pathways,^73^ which may help explain their superior efficacy when used in combination. In HIV, preliminary data also suggested a greater effect on reversing latency with combined blockade of PD-1 and CTLA-4 compared to anti-PD-1 alone,^67^ but it remains unclear whether this is mediated through additive or synergistic effects. Immunological and genetic profiling revealed distinct genomic and functional signatures of combined blockade compared to either therapy alone, which may suggest a potential for immunological synergism.^74^ It is also possible that engagement of distinct T-cell populations by anti-CTLA-4 and anti-PD-1 contributes to the enhanced effect of combination blockade, as was indicated by the differential expression of PD-1 and CTLA-4 on resting and proliferating CD4+ T including findings that anti-PD-1 could reverse latency in non-proliferating cells and anti-CTLA-4 in proliferating T-cells.^33^

We recently explored the effect on HIV-specific T-cell function of multiple combinations of immune checkpoint antibodies *ex vivo* using PBMCs obtained from PWH on suppressive ART. This showed that immune checkpoint blockade primarily led to enhanced production of CD107a and IL-2 but not IFNγ and TNFα in response to HIV peptide stimulation.^75^ Combinations that included antibodies to LAG-3, CTLA-4 and TIGIT showed synergistic induction of cytokine production in HIV-specific T-cells, whereas combinations that included anti-PD-1 did not.^75^ Another study using PBMCs from PWH on ART evaluated antibodies to CTLA-4, TIM-3, LAG-3, CD160 and BTLA, alone or in combination with anti-PD-1, and found that anti-TIM-3 and anti-BTLA enhanced CD8+ T-cell proliferation in response to HIV peptide stimulation.^76^

Taken together, these studies indicate enhanced effect and potential synergy of combining blockade against several immune checkpoints, but much more work is required to understand which pathways should ideally be targeted to promote elimination of latently infected cells and, secondly, how such combinations might be administered without excessive toxicity.

## Outstanding questions

Several important questions remain to be answered for a more comprehensive understanding of the potential for using ICB as a therapeutic tool in achieving ART-free control of HIV. First, safety remains a significant concern as illustrated by the premature termination due to immune-related adverse events (irAEs) of two clinical trials in HIV using anti-PD-1 and anti-PDL1.^77^^,^^78^ These experiences raise a clear need for more effective risk mitigation strategies. Given that current data is derived from blocking PD-1/PD-L1 or CTLA-4, emerging clinical trial data of antibodies against LAG-3, TIM-3 and TIGIT will be highly informative when considering their safety and potential use in HIV. Aligned with results from cancer treatment, preliminary data indicate that only a subset of treated PWH will respond to and profit from ICB. In people with malignancies, multiple mechanisms of primary and secondary resistance to ICB have been recognised. While many of these are changes that originate in the tumour cells and which impair antitumour immune responses, others are host-related including alterations in immunosuppressive cells, secretion of cytokines and chemokines, composition of the gut microbiome, and co-expression of multiple inhibitory immune checkpoints.^79^ For example, in patients with non-small cell lung cancer, high expression of PD-1 marked a particularly dysfunctional subset of T-cells characterised by co-expression of multiple other immune checkpoints and was associated with poor restoration of T-cell effector function following PD-1 blockade.^80^ The prediction of who can mount a sustained HIV-specific T-cell response to ICB and which mechanisms underlie such a treatment-response remains to be determined, but may be informed from host-specific findings in cancer immunotherapy and will be of tremendous value for the design of novel curative strategies. It may also be possible to mitigate risk through single and/or low-dose administration of immune checkpoint antibodies. For example, a study in people with hepatitis B demonstrated that a single low-dose (0.1 or 0.3 mg/kg) of the anti-PD-1 antibody, nivolumab, was safe and led to high levels of PD-1 occupancy for around 6 weeks.^81^

Second, most immune checkpoints are also expressed on immune cells other than CD4+ and CD8+ T-cells such as NK cells, monocytes, gamma delta T-cells and Tregs. These cells are important to consider as additional targets and the effect of blocking immune checkpoints in those cell populations needs to be better understood.^82^ For example, blocking a co-inhibitory signal on cytotoxic CD8+ T-cells can simultaneously act as a co-stimulatory signal in suppressive Tregs, which then can lead to overactivation-induced Treg apoptosis and result in elimination of neative Treg mediated suppression. However, targeting immune checkpoints on Tregs can also potentiate TCR stimulation, which does not result in overactivation-induced apoptosis, but instead enhances Treg proliferation^83^ thereby increasing unwanted Treg mediated suppression.

Third, given that immune checkpoints provide co-inhibitory or co-stimulatory signals to T-cells upon binding of their cognate antigen, it is entirely possible that blocking inhibitory receptors during antigen exposure may provide a more potent immune stimulus. This will require studies delivering ICB in the setting of ART interruption as HIV antigen expression is minimal on ART.

Fourth, data from animal models show that the crystallizable fragment (Fc) variant of the immune checkpoint targeting antibody has a profound impact on the antitumor response.^84^ In the case of anti-CTLA-4, the Fc tail was shown to have a critical role in promoting antibody-mediated cellular cytotoxicity (ADCC) leading to an increased CD8^+^ to Treg ratio, which promoted tumour rejection.^85^^,^^86^ However, the translational implications of these findings have thus far not been investigated in the setting of HIV.

Fifth, accumulating data on inhibitory pathways and their modulation in HIV can form the basis of using ICB as a vaccine adjuvant. In animal models, ICB during vaccination improved protective efficacy**^36^** and increased levels of anti-HIV antibodies with higher antibody-dependent cellular cytotoxicity capabilities.^44^

Finally, while most studies have focused on blocking negative receptors, there are emerging data on agonistic or modulatory interactions with co-stimulatory pathways. The co-stimulatory glucocorticoid-induced TNFR related protein (GITR, also known as TNFRSF18) is a potent target for immunotherapy in mouse models of cancer and chronic infection, owing to its capacity to concurrently promote effector T-cell function and dampen Treg-mediated suppression. A first-in-human trial in patients with solid tumours demonstrated safety of a monoclonal antibody targeting GITR but failed to demonstrate clinical efficacy or clear reinvigoration of exhausted T-cells.^87^ Investigating co-stimulatory agents like GITR and CD226 in the context of HIV might still hold potential as “putting the gas on” rather than “taking the brakes off” might overcome exhaustion in chronic infection. In a vaccination setting, co-stimulatory ligands could also boost the priming of immune cells and facilitate development of an effective and sustained anti-HIV immune response. More work is required to investigate these potentially promising pathways.

## Conclusion

In conclusion, a substantial body of pre-clinical data highlights the potential of ICB for targeting persistent SIV and HIV infection. Clinical data derived from PWH on ART with or without malignancy indicate a modest effect of reversing HIV latency with the clearest effects to date seen in individuals receiving combination blockade that included anti-CTLA-4. To which extent and by which mechanisms ICB can durably enhance HIV specific T-cell function is still being explored, but preliminary data indicate this may occur only in a subset of treated individuals. This is consistent with results from cancer treatment where anti-tumour response rates with anti-PD-1 range as widely as 4–70% depending on tumour pathology.^88^

While targeting more than one inhibitory pathway may be superior to single-agent blockade, the enhanced effect may come at a cost of increased toxicity and it is currently unclear how these findings can be translated into clinical strategies for HIV cure. The high and rapidly expanding number of ongoing clinical trials investigating combination approaches in people with malignancies will provide further information on whether combined blockade can improve clinical efficacy relative to toxicity but given the excellent prognosis for PWH on ART, there is a much lower tolerance for immune-related toxicities in this population. Single or combined blockade of immune checkpoints in HIV is therefore primarily focused on understanding how interrupting the negative signalling, or potentiating co-stimulatory signalling, might contribute to augmenting HIV-specific T-cell function and/or eliminating latently infected CD4+ T-cells. With future improvements in risk mitigation or novel approaches to deliver ICB safely to PWH without cancer, combinations of immune checkpoint antibodies may become a viable clinical strategy to test in HIV cure studies.

## Search strategy and selection criteria

We searched the PubMed and Clarivate database in August 2021 with the keywords: PD-1, PD-L1, PD-L2, CTLA-4, TIGIT, TIM-3, LAG-3, GITR, anti-PD-1, anti-PD-L1, anti-PD-L2, anti-CTLA-4, anti-TIGIT, anti-TIM-3, anti-LAG-3, anti-GITR and immune checkpoints in combination with HIV or SIV. Titles and abstracts were reviewed and assessed for relevance.

## Declaration of interests

Dr. Lewin reports grants from National Health and Medical Research Council (NHMRC), grants from the National Institutes of Health (NIH), grants from the Australian Centre for HIV and Hepatitis (ACH2), during the conduct of the study; grants from amFAR, grants and personal fees from Gilead Sciences, grants and personal fees from Merck, grants from Melbourne HIV Cure Consortium, grants from Wellcome Trust, grants from NFACR, other from Callimune, personal fees and other from Abivax, personal fees from Geovax, personal fees and other from ViiV, personal fees from Tetralogic, personal fees from Bristol Myers Sqibb, other from Bionor, other from InniVirVax, other from Aelix Therapeutics, personal fees and other from Immunocore, other from Vaxxinity, grants from Leidos, personal fees from Esfam, other from ANRS Emerging Infectious Diseases, grants from NHMRC CRE, grants from NHMRC partnership project, outside the submitted work; In addition, Dr. Lewin has a patent International PCT patent (PCTAU2017050631) pending. Dr. Rasmussen reports grants from Melbourne HIV Cure Consortium, grants from ACH2, grants from Region Midt Denmark, outside the submitted work. Dr. Gubser reports grants from Novartis Research fellowship, grants from NHMRC, grants from ACH2, other from Millenium Science, grants from Doherty Grant scheme, other from ViiV, outside the submitted work. Dr. Chiu has nothing to disclose.
